# Motion compensated cine CMR of the fetal heart using radial undersampling and compressed sensing

**DOI:** 10.1186/s12968-017-0346-6

**Published:** 2017-03-20

**Authors:** Christopher W. Roy, Mike Seed, John C. Kingdom, Christopher K. Macgowan

**Affiliations:** 1 Department of Medical Biophysics, University of Toronto, Toronto, ON Canada; 2 Division of Physiology and Experimental Medicine, The Hospital for Sick Children, Toronto, ON Canada; 3 Division of Pediatric Cardiology, The Hospital for Sick Children, Toronto, ON Canada; 4 Departments of Pediatrics and Diagnostic Imaging, University of Toronto, Toronto, ON Canada; 5 Department of Obstetrics and Gynaecology, Mount Sinai Hospital, Toronto, ON Canada; 6 Department of Obstetrics and Gynaecology, University of Toronto, Toronto, ON Canada

**Keywords:** Fetal CMR, Golden angle radial, Motion correction, Post-processing, Compressed sensing, Accelerated imaging

## Abstract

**Background:**

To develop and evaluate a reconstruction framework for high resolution time-resolved CMR of the fetal heart in the presence of motion.

**Methods:**

Data were acquired using a golden angle radial trajectory in seven fetal subjects and reconstructed as real-time images to detect fetal movement. Data acquired during through-plane motion were discarded whereas in-plane motion was corrected. A fetal cardiac gating signal was extracted to sort the corrected data by cardiac phase, allowing reconstruction of cine images. The quality of motion corrected images and the effect of data undersampling were quantified using separate expressions for spatial blur and image error.

**Results:**

Motion corrected reordered cine reconstructions (127 slices) showed improved image quality relative to both uncorrected cines and corresponding real-time images across a range of root-mean-squared (RMS) displacements (0.3–3.7 mm) and fetal heart rates (119–176 bpm). The relative spatial blur between cines with and without motion correction increased with in-plane RMS displacement leading to an effective decrease in the effective spatial resolution for images without motion correction. Image error between undersampled and reference images was less than 10% for reconstructions using 750 or more spokes, yielding a minimum acceptable scan time of approximately 4 s/slice during quiescent through plane motion.

**Conclusions:**

By rejecting data corrupted by through-plane motion, and correcting data corrupted by in-plane translation, the proposed reconstruction framework accounts for common sources of motion artifact (gross fetal movement, maternal respiration, fetal cardiac contraction) to produce high quality images of the fetal heart.

**Electronic supplementary material:**

The online version of this article (doi:10.1186/s12968-017-0346-6) contains supplementary material, which is available to authorized users.

## Background

Dynamic cardiovascular magnetic resonance (CMR) of the fetal heart requires a combination of clinically relevant spatial resolution, temporal resolution, signal-to-noise-ratio (SNR), and volumetric coverage. During late gestation, the size of major fetal vessels is in the range of 5–10 mm in diameter, the width of the fetal ventricles is in the range of 10–30 mm [1], and the duration of the fetal cardiac cycle is between 110 and 180 bpm [2, 3]. As a result, spatial and temporal resolutions on the order of 1 mm and 30 ms respectively are required to define fetal cardiac anatomy during dynamic motion [4]. This task is challenging because the effective resolution and overall quality of fetal images is often degraded by gross fetal movement and un-gated periodic motion (fetal cardiac motion and maternal respiration). Currently available methods developed to compensate for these degrading effects include motion-tolerant k-space trajectories [5–7], assessment and correction of motion [8–11], cardiac and respiratory gating, [6, 8, 12–14], and accelerated imaging [6, 14, 15].

In this work, we develop and evaluate a novel framework for reconstructing high spatial and high temporal resolution time-resolved images of the fetal heart in the presence of motion, based on a combination of established methods for cardiac imaging. First, fetal data are acquired using a continuous golden angle radial trajectory and static images, reconstructed from all acquired spokes for each slice, are used to confirm slice prescription and provide an initial assessment of fetal activity. Radial trajectories are appealing because they are less sensitive to motion artifact, compared to conventional Cartesian sampling, due to temporal oversampling of the k-space origin [16]. Second, real-time reconstruction of the data enables assessment and correction of both periodic and stochastic motion. This is accomplished by manually rejecting data acquired during periods of through-plane motion [6, 14, 17], correcting the remaining data for translational in-plane motion [18, 19], and extracting cardiac gating signals using metric optimized gating [12, 20]. Both real-time and reordered cine reconstructions of the data rely on compressed sensing to suppress streaking artifacts from radial undersampling [5, 6, 21]. The resulting cine reconstructions combine data from multiple heart beats to provide improved spatial resolution, temporal resolution and SNR [19].

The primary aims of this work are to evaluate the proposed methods for motion-robust imaging of the fetal heart, and to determine the minimum scan time (per slice) that provides clinically acceptable spatial resolution, temporal resolution and signal-to-noise-ratio for fetal cardiac applications. These aims will be accomplished by qualitatively and quantitatively comparing reconstructions with and without motion correction, and across a range of acceleration factors, using fetal CMR scans.

## Methods

### Fetal CMR acquisition

A steady state free precession sequence with continuous golden angle radial sampling was used to acquire data from human fetuses in the third trimester. Slices were prescribed in both short-axis and long-axis views on a 1.5 T clinical MRI system using both body and spine matrices with approximately thirty active channels (Avanto^Fit^, Siemens Healthcare – Germany). For each view, 10–15 slices were acquired spanning the heart. Seven pregnant women (normal fetal cardiac anatomy = 3, congenital heart disease = 4, all at gestational ages 34–38 weeks) were enrolled for this IRB approved prospective study and written consent to acquire and publish data was obtained from each subject. Fetal radial acquisitions were performed for 5 min, as part of a larger one-hour examination. All scans were performed free-breathing with the following acquisition parameters: flip angle: 70°, acquired spokes: 3000, TR: 4.95 ms, samples per spoke: 256 (with two-fold oversampling to avoid wrap), field-of-view: 256 × 256 mm^2^, spatial resolution: 1 × 1 × 4 mm^3^, and scan length: ~15 s per slice.

### Reconstruction of real-time images

**Fig. 1:**
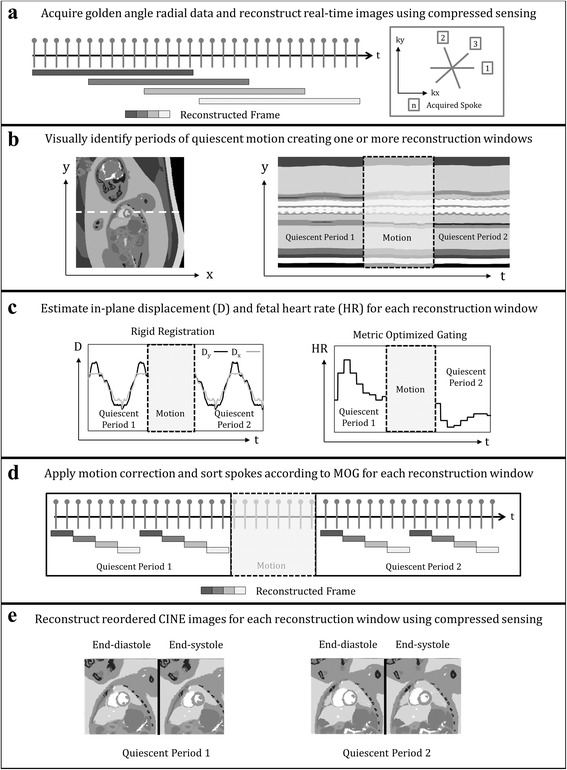
Diagram outlining the proposed framework for reconstructing images of the fetal heart in the presence of motion using a golden angle radial acquisition (**a**), real-time reconstruction to assess and reject through-plane motion (**b**), estimate in-plane motion and fetal heart rate (**c**), correct and sort k-space (**d**), and reconstruct reordered cine images (**e**). Further details for each of these steps is provided in the methods section

Figure 1a illustrates the golden angle trajectory, where each new radial spoke divides the largest angular gap of the preceding set [16]. The advantage of this gap filling approach is that a relatively uniform (though sub-Nyquist) sampling of k-space can be obtained with only a small number of radial spokes, facilitating real-time reconstruction of the data. Real-time reconstructions were performed using a 15-spoke sliding window (Fig. 1a), resulting in a real-time temporal resolution of ~74 ms, interpolated to ~25 ms using 10 shared spokes between frames (~600 frames per real-time image series).

The number of spokes used to reconstruct each real-time frame was well below the Nyquist sampling criterion (acceleration factor of 27) and therefore compressed sensing was used to supress aliasing artifacts. Based on previous fetal CMR work with compressed sensing, three image regularization terms were used: spatial total variation, temporal total variation, and the Fourier transform applied along the temporal dimension [15]. Applying these parameters to real-time reconstructions allowed for subsequent assessment and correction of motion, and extraction of fetal cardiac gating signals.

### Motion compensation

Motion compensation was performed in two steps. First, real-time images were assessed visually to identify one or more periods of negligible through-plane motion (Fig. 1b). While this step may be automated [22], it was performed manually in this work, aided by line-plots of non-dynamic fetal anatomy versus time (i.e., lungs, liver). Second, data undergoing visually noticeable through-plane motion were discarded while data from each identified quiescent period were independently reconstructed and in-plane motion was quantified using translational motion estimation over a region of interest containing the fetal heart and surrounding anatomy. Briefly, the region of interest in each real-time frame was iteratively translated in-plane and compared, by mutual information, to a target frame representing the mean of all images within the given quiescent period [23, 24]. The result of this iterative registration routine was a motion corrected real-time image series and two vectors describing the in-plane displacement of the fetal abdomen versus real-time frame (Fig. 1c). Motion-correction of the original K-space data was then performed by interpolating the displacement vectors to match the timing of each K-space spoke, and applying the Fourier shift theorem. Using the motion corrected real-time images, a cardiac gating signal was extracted using metric optimized gating, which can subsequently be used to reorder the motion-corrected K-space according to cardiac phase.

### Metric Optimized Gating (MOG)

MOG is a retrospective gating technique for determining the cardiac phase of CMR data when an ECG signal is unavailable. This approach has been previously validated in postnatal subjects and applied to dynamic fetal imaging, including integration with compressed sensing [12, 15, 20]. In this work, MOG was applied to the motion-corrected real-time images in the following manner. First, an estimation of the fetal heart rate was used to retrospectively combine real-time frames according to cardiac phase. Second, an image metric (entropy), was calculated over a region of interest containing the heart. Third, iterative minimization of the image metric was performed to optimize the initial estimation of fetal heart rate and synthesize a cardiac gating signal akin to the fetal ECG (Fig. 1c).

### Reconstruction of reordered CINE images

Using the MOG gating signal, the motion-corrected K-space data were binned according to cardiac phase (Fig. 1d) and combined to produce cine images of the fetal heart (Fig. 1e). Reordered cine images were reconstructed to 30 frames without view sharing, resulting in an effective temporal resolution of 12–15 ms depending on the fetal heart rate. The number of spokes per reordered cine frame varied between 25 and 100 (acceleration factors between 4 and 16) depending on the number of spokes rejected due to through-plane motion. As a result, compressed sensing was again used to suppress undersampling artifacts, with the same image regularization terms as described for real-time reconstruction.

### Evaluating motion compensation

The intended result of motion compensation is to increase spatial sharpness in reordered cine images. However, it is inherently difficult to quantify and compare the level of spatial sharpness among images from different slice positions or volunteers [25]. As a result, in addition to qualitative evaluation, reordered cine reconstructions pre and post motion correction were quantitatively compared using a metric for relative spatial blur derived as follows [26–28]. First, motion corrected images were filtered with a two-dimensional spatial Gaussian smoothing kernel with variable standard deviation. Second, the standard deviation of the kernel was varied and the filtered cine images were compared (over a region of interest) to the corresponding uncorrected images. This comparison was made using the normalized root-mean-squared difference between the two series:

1 $$ \mathrm{Image}\ \mathrm{Error}=100\ast \sqrt{\frac{{\displaystyle {\sum}_{\mathrm{t}}}{\displaystyle {\sum}_{\mathrm{i}}}{\left|{\mathrm{m}}_{\mathrm{i},\mathrm{t}}-{\mathrm{r}}_{\mathrm{i},\mathrm{t}}\right|}^2}{{\displaystyle {\sum}_{\mathrm{t}}}{\displaystyle {\sum}_{\mathrm{i}}}{\left|{\mathrm{r}}_{\mathrm{i},\mathrm{t}}\right|}^2}} $$

where m_i,t_ represents the filtered motion corrected images, r_i,t_ represents the original uncorrected images, and image error is calculated over spatial locations, i, and cardiac phases, t [29]. Spatial blur due to in-plane motion was then defined as the standard deviation of the Gaussian smoothing kernel, normalized to pixel size (mm), that minimized the image error between reordered cine reconstructions pre and post motion correction.

To compare spatial blur between different volunteers, the level of in-plane motion for a given acquisition was calculated using the root-mean-square displacement (D_RMS_):

2 $$ {D}_{RMS} = \sqrt{\frac{1}{T}{{\displaystyle {\sum}_{t=1}^T\left({D}_x(t)-{\overline{D}}_x\right)}}^2+{\left({D}_y(t)-{\overline{D}}_y\right)}^2} $$

where D_x_ and D_y_ are the horizontal and vertical components of displacement calculated from real-time frames (t) as described by Fig. 1c.

### Evaluating compressed sensing

To assess the ability of compressed sensing to supress radial streaking artifact in reconstructions of the fetal heart, and to determine the minimum acceptable scan time per slice, fetal datasets with a reduced number of spokes (N_R_) were retrospectively generated by truncating the original acquisitions. To ensure that the range of motion was conserved at different undersampling factors, and so did not influence this analysis, only acquisitions with negligible through-plane motion were used. Furthermore, truncated acquisitions were chosen such that the D_RMS_ matched that of the original full acquisition.

In addition to qualitative evaluation, undersampled acquisitions were quantitatively compared using the same expression for image error (Eq. 1), but comparing each undersampled dataset m_i,t_ (N_R_ = 250, 500, 750, 1000, 1500, 2000, 2500) to a reference dataset r_i,t_ (N_R_ = 3000). Given that the differences between m_i,t_ and r_i,t_ stem from the cumulative effect of image regularization (spatial and temporal), residual aliasing, and random noise, image error in this context is a global measure of reconstruction artifact.

### Image reconstruction and post-processing

All real-time and reordered cine reconstructions and subsequent analyses were performed on a personal computer with a single i7-6700 processor (Intel Corporation, Santa Clara, California, USA; clock speed 2.60 GHz; 4 cores) and 32 GB of RAM. CS was implemented using a conjugate gradient algorithm coded in MATLAB (MathWorks - Natick, MA, USA) [21, 30]. The relative weights for the CS regularization terms were determined by retrospectively undersampling a fetal data set without visible through-plane or in-plane motion, and choosing values that minimized image error as given by Eq. (1). Regularization weights of 0.0025, 0.025, and 0.025 were chosen for spatial total variation, temporal total variation, and temporal Fourier transform, respectively, and then applied to all subsequent reconstructions. The CS algorithm ran for 50 iterations per slice resulting in CS reconstruction times of 120 min and 15 min for real-time and reordered cine images respectively.

Registration based on the mutual information of real-time reconstructions was performed using open source elastix software [23, 24]. The optimization routine typically ran for 250 iterations per frame resulting in a processing time of 10–15 min per slice.

The MOG algorithm used in this work is based on a previously published multistep minimization routine that uses a steepest descent gradient search, also coded in MATLAB [15]. MOG processing time was typically less than one minute per image series resulting in a combined processing time for real-time CS reconstruction, motion correction, MOG, and reordered cine CS reconstruction of approximately 2.5 h per slice including <5 min per slice to manually identify through plane motion and regions of interest containing the heart.

## Results

Table 1 summarizes the analysis of real-time images across all seven pregnant volunteers. For each volunteer 20–25 slices (N_tot_) were acquired to span the fetal heart in both short-axis and long-axis views. A total of 150 slices were acquired across all volunteers, of which 127 (N_card_) contained cardiac anatomy. Of the slices that contained cardiac anatomy, visual assessment of real-time cardiac images identified 96 slices (76% of N_card_) with only in-plane motion (N_xy_) and 31 slices (24% of N_card_) with both in-plane and through-plane motion (N_xyz_), recognizing however that detection of in-plane motion is inherently more sensitive (in-plane resolution: 1×1 mm^2^) than through-plane (slice thickness: 4 mm). All acquisitions, regardless of the level of motion, contained at least one visually identified quiescent period suitable for subsequent analysis, with a mean duration of quiescent periods (T_Q_) across all volunteers of 5.5 s (range: 1.3–12.4 s). Image registration of real-time images yielded estimations of in-plane translational displacement of the heart, with a mean RMS displacement (D_RMS_) across all volunteers of 1.2 mm (range 0.3–3.7 mm). Extraction of fetal cardiac gating signals using MOG yielded a mean heart rate of 144 bpm (range 119–176 bpm), which is consistent with the normal fetal cardiac cycle (110–180 bpm) [2, 3].

**Table 1:** Overview of fetal real-time image analysis for each volunteer

	Slice counts				Motion parameters		
ID	N_tot_	N_card_	N_xy_	N_xyz_	T_Q_ (s)	D_RMS_ (mm)	HR (bpm)
1	20	16	11	5	3.3 (1.5–5.0)	2.5 (1.6–3.7)	140 (128–161)
2	20	17	17	0	-	0.5 (0.3–0.8)	145 (134–166)
3	20	15	13	2	7.1 (3.0–11.7)	1.5 (0.8–2.0)	131 (119–145)
4	25	25	18	7	7.0 (3.8–11.2)	1.5 (1.0–2.3)	152 (130–168)
5	20	17	5	12	4.8 (1.3–12.4)	0.8 (0.6–1.1)	169 (166–170)
6	25	22	18	4	5.1 (2.5–10.6)	1.0 (0.4–1.9)	132 (125–143)
7	20	15	14	1	6.6 (3.8–12.4)	1.0 (0.5–1.7)	159 (146–176)

*ID* Volunteer identification, *N*_*tot*_ Number of slices acquired, *N*_*card*_ Number of slices containing cardiac anatomy, *N*_*xy*_ Number of slices containing cardiac anatomy with only in-plane motion, *N*_*xyz*_ Number of slices containing cardiac anatomy with both in-plane and through-plane motion, *T*_*Q*_ Duration of quiescent through-plane motion, *D*_*RMS*_ Root-mean-square of in-plane displacement, *HR* Fetal heart rate. quiescent period, displacement and heart rate values represent the mean and range

**Fig. 2:**
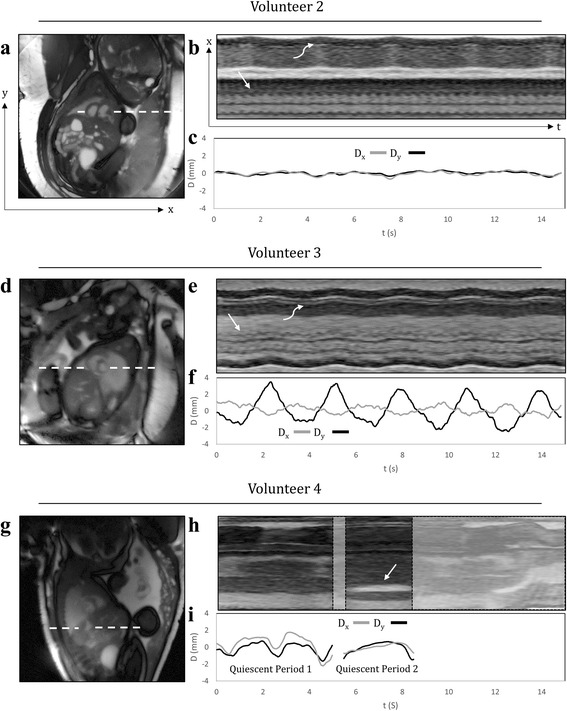
Real-time reconstructions of the fetal heart for three representative volunteers with increasing levels of motion. Cine movies corresponding to these reconstructions are available as supporting video S1. Time-averaged images of the heart and surrounding anatomy (**a**, **d**, **g**), M-mode displays of signal along the white dashed lines (**b**, **e**, **h**), and plots of displacement (D) versus time (**c**, **f**, **i**), calculated from the real-time reconstructions over a region of interest containing the heart. D_X_ and D_Y_ represent the horizontal and vertical components of displacement, respectively. For volunteers two and three, the M-mode displays show fetal cardiac motion (straight arrow) and maternal respiratory motion (curved arrow). For volunteer four, the M-mode display shows the fetal stomach (straight arrow), which is used to identify through-plane motion



Figure 2 displays representative results from real-time reconstructions of the fetal heart. Short-axis views are shown for three subjects with increasing levels of motion with cine videos of these reconstructions provided by supplementary video 1. These data correspond to volunteers 2, 3 and 4 from Table 1, respectively. For each subject, time-averaged reconstructions depict the fetal and maternal anatomy (Fig. 2a, d, g). Line plots of signal intensity versus time (M-mode display) depict the temporal dynamics of the acquisition, including fetal cardiac motion and maternal respiration (Fig. 2b, e), and gross fetal movement (Fig. 2h). Finally, plots of translational displacement (D) versus time depict in-plane motion (Fig. 2c, f, i).

**Additional file 1: Video S1.** Real-time reconstructions of the fetal heart for three representative volunteers with increasing levels of motion. This video corresponds to the same reconstructions shown in Fig. 2. a) Minor displacement of the heart. b) Noticeable in-plane displacement of the heart due to maternal respiration. c) Noticeable through-plane and in-plane motion due to maternal respiration and gross fetal movement. (MP4 14763 kb)


In the first example (Fig. 2a-c), through-plane and in-plane displacements of the fetal heart were small, and fetal cardiac motion was well visualized across the entire acquisition window. In the second example (Fig. 2d-f), the fetal heart was periodically displaced by maternal respiration. However, cardiac contraction was visible throughout the acquisition window because through-plane motion was negligible. In the third example (Fig. 2g-i), the presence of gross fetal movement resulted in both through-plane and in-plane displacement of the fetal heart. To better identify through-plane displacement and periods of quiescent fetal movement, an M-mode display (Fig. 2h) through normally static fetal tissues, such as the liver and stomach, was used. In this example, two quiescent periods of fetal movement were identified for subsequent analysis of in-plane displacement and reconstruction.

### Evaluating motion compensation

**Fig. 3:**
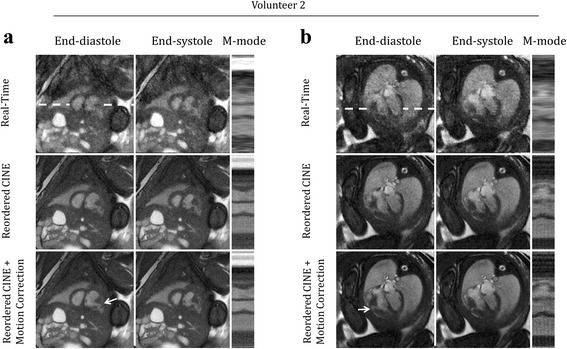
Qualitative evaluation of motion compensation. Representative real-time and reordered cine reconstructions (with and without motion correction) of fetal hearts undergoing minor displacement. Cine movies corresponding to these reconstructions are available as supporting video S2. Short axis (**a**) and long axis (**b**) views are shown at end-diastole, end-systole and as M-mode displays of signal along the white dashed lines. For comparison, one representative cycle from the real-time reconstructions is shown. Arrows denote a right ventricle diverticulum that is best visualized by the reordered cine images



Figure 3 displays reconstructions of the fetal heart in short-axis (Fig. 3a) and long-axis (Fig. 3b) views from volunteer 2 with cine videos of these reconstructions provided by supplementary video 2. For comparison, a representative heart beat from the real-time reconstruction is shown along with cine reconstructions before and after motion correction. Images of end-diastole and end-systole are shown, along with an M-mode display of fetal cardiac dynamics. In the real-time views (Fig. 3, top), myocardial contraction was visualized. However, image quality was noticeably higher in the reordered cine images, with reduced noise and temporal blurring relative to the real-time reconstructions. As a result, cine images improved visualization of cardiac structures, such as the congenital defect depicted in Fig. 3 (white arrows: right ventricular diverticulum). For this volunteer, motion correction provided only subtle improvements in image quality because displacements were small (Fig. 3a D_RMS_: 0.4 mm, Fig. 3b D_RMS_: 0.6 mm).

**Additional file 2: Video S2.** Representative reconstructions of fetal hearts undergoing minor displacement. This video corresponds to the same images shown in Fig. 3. For comparison, one representative cycle from the real-time reconstructions is shown, as well as reordered cine reconstructions (with and without motion correction) in short axis (a) and long axis (b) views. Arrows denote a right ventricle diverticulum that is best visualized by the reordered cine images. (MP4 10531 kb)


**Fig. 4:**
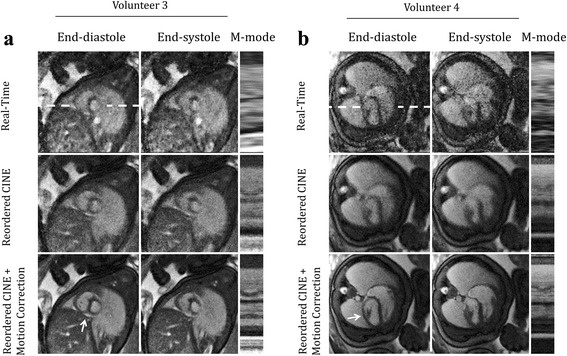
Qualitative evaluation of motion compensation. Representative real-time and reordered cine reconstructions (with and without motion correction) of fetal hearts undergoing in-plane motion. Cine movies corresponding to these reconstructions are available as supporting video S3. Short axis (**a**) and long axis (**b**) views are shown at end-diastole, end-systole and as M-mode displays of signal along the white dashed lines. For comparison, one representative cycle from the real-time reconstructions is shown. Arrows denote a thin rim of pericardial fluid (**a**) and left ventricular anterior papillary muscle (**b**) which are best visualized by the reordered cine images



Figure 4 displays fetal images acquired during more dramatic in-plane motion for short axis (volunteers 3) and long-axis (volunteer 4) views with cine videos of these reconstructions provided by supplementary video S3. Interestingly, the real-time reconstructions appear spatially sharper than the reordered cine images before motion correction, albeit with increased noise and artifact. This is due to the level of in-plane motion that occurred during the scans (Fig. 4a D_RMS_: 1.6 mm, Fig. 4b D_RMS_: 1.9 mm) which is resolved by the real-time reconstructions but contributes blur to reordered cine images without motion correction. As a result, cine image sharpness was visibly improved by motion correction, with fine cardiac structures better resolved. For example, a thin rim of pericardial fluid and right ventricular trabeculation were well defined after motion correction (arrows in Fig. 4a and b, respectively).

**Additional file 3: Video S3.** Representative reconstructions of fetal hearts undergoing in-plane motion. This video corresponds to the same images shown in Fig. 4. For comparison, one representative cycle from the real-time reconstructions is shown, as well as reordered cine reconstructions (with and without motion correction) in short axis (a) and long axis (b) views. Arrows denote a thin rim of pericardial fluid (a) and right ventricle trabeculation (b) which are best visualized by the reordered cine images. (MP4 12361 kb)


**Fig. 5:**
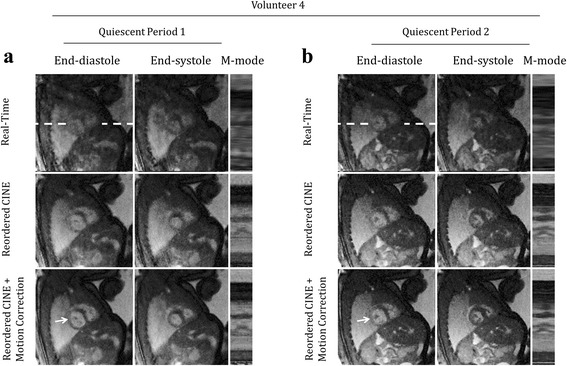
Qualitative evaluation of motion compensation. Representative real-time and reordered cine reconstructions of fetal hearts undergoing through plane motion. Cine movies corresponding to these reconstructions are available as supporting video S4. Short axis views derived from separate reconstruction of quiescent periods (**a** and **﻿b**) are shown at end-diastole, end-systole and as M-mode displays of signal along the white dashed lines. For comparison, one representative cycle from the real-time reconstructions is shown for each quiescent period. Papillary muscles are denoted by arrows and are best visualized by the reordered cine images



Figure 5 displays fetal images acquired during both through-plane and in-plane motion in a short-axis view from volunteer 4 with cine videos of these reconstructions provided by supplementary video S4. Two quiescent periods of 5.8 s (Fig. 5a) and 2.9 s (Fig. 5b) were identified and reconstructed in the manner described by Fig. 1 and each quiescent period provided different slice positions due to through-plane motion. Over both periods, radial contraction was well visualized in the reordered cine reconstructions, as were papillary muscles in the left ventricle (arrows in Fig. 5a, b). Again, spatial sharpness qualitatively improved after motion correction due to the level of in-plane motion that occurred during the scan (Fig. 5a D_RMS_: 0.7 mm, Fig. 5b D_RMS_: 1.0 mm). Further analysis of in-plane motion and its impact on cine image quality is provided below using a quantitative metric for image blur.

**Additional file 4: Video S4.** Representative reconstructions of fetal hearts undergoing through-plane motion. This video corresponds to the same images shown in Fig. 5. For comparison, one representative cycle from the real-time reconstructions is shown for each quiescent period as well as well as reordered cine reconstructions (with and without motion correction) in short axis views derived from separate reconstruction of quiescent periods (**a** and **b**). Papillary muscles are denoted by arrows and are best visualized by the reordered cine images. (MP4 8384 kb)


**Fig. 6:**
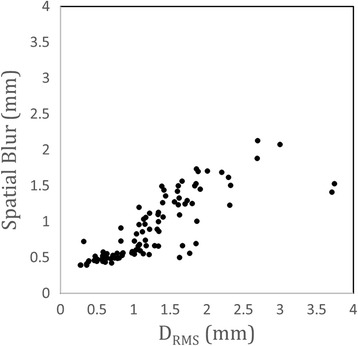
Quantitative evaluation of motion compensation. Spatial blur, as defined in the Methods section, versus RMS displacement (D_RMS_) as given by Eq. 2 for all acquisitions undergoing only in-plane motion (N_xy_ = 96). For in-plane displacements greater than the acquired pixel size (1 mm^2^) the effective spatial resolution is significantly decreased

Figure 6 plots the spatial blur of reordered cine images without motion correction versus RMS displacement (D_RMS_) as given by Eq. 2. For this analysis, only acquisitions without visible through-plane motion were included (N_RMS_ = 96). As expected, spatial blur increases with increasing displacement resulting in a significantly worse effective spatial resolution for uncorrected images undergoing displacements greater than the acquired spatial resolution (1 mm). Variability in spatial blur between slices with similar levels of mean displacement may be attributed to discrepancies between the spatial blur metric, which assumed Gaussian blur, and the more complex underlying motion which may include through-plane motion.

Still these quantitative results agree with the qualitative examples shown in Figs. 3, 4 and 5 where displacement values greater than 1 mm resulted in visually noticeable increases in spatial blur. These quantitative results, combined with the qualitative results from Figs. 3, 4 and 5, demonstrate the need for motion compensation to preserve spatial resolution in fetal CMR.

### Evaluating compressed sensing

**Fig. 7:**
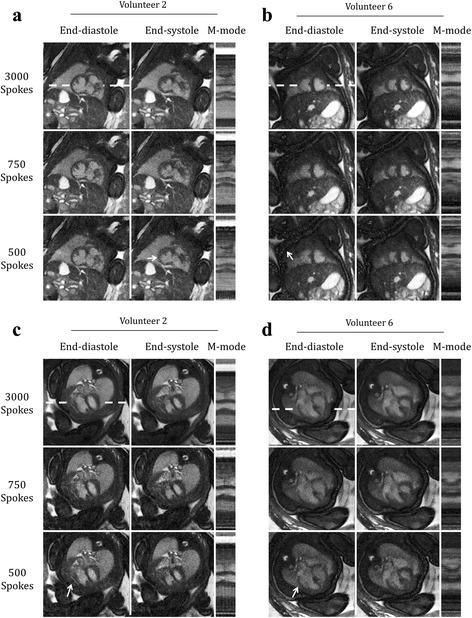
Qualitative evaluation of compressed sensing. Reordered cine reconstructions of four retrospectively undersampled data sets (number of reconstructed spokes (N_R_) = 500, 750, 3000), shown at end-diastole, end-systole and as M-mode displays of signal along the white dashed lines. Cine movies corresponding to these reconstructions are available as supporting video S5. Short axis (**a** & **b**) and long axis (**c** & **d**) views are shown for two different volunteers. Arrows denote the thickness of the myocardium (**a**), fetal abdominal cavity (**b**), and pericardium (**c** & **d**), features that are degraded with increasing undersampling



Figure 7 displays retrospectively undersampled acquisitions used to evaluate the compressed sensing component of the reconstruction framework. Four representative examples, including short axis (Fig. 7a-b) and long axis (Fig. 7c-d) views, illustrate the effects of undersampling on a variety of fetal cardiac structures with cine videos of these reconstructions provided by supplementary video S5. Reconstructions of 3000, 750 and 500 spokes are shown corresponding to undersampling factors (with respect to the Nyquist criterion) of 4, 16 and 24 respectively. Visualization of major cardiac structures was generally preserved across the tested undersampling factors, with reconstructions from 750 spokes providing an acceptable trade-off between image quality and reconstruction artifact, compared to those from 3000 spokes. However, image degradation was apparent when using only 500 spokes. For example, in Fig. 7a the border between the myocardium and blood pool (arrow in Fig. 7a end-systole) appears blurred when comparing reconstructions of 500 and 3000 spokes. Similarly, horizontal lines in the M-mode display for 500 spokes are indicative of blur from regularization. Residual aliasing is best visualized by the cine videos (supporting video S5), however it also appears as vertical lines in the M-mode display and increases with fewer spokes (Fig. 7a). In Fig. 7b, increased noise combined with regularization resulted in noticeable structured artifact in the fetal abdominal cavity (arrow in Fig. b end-diastole). Again, blur is evident as horizontal lines in the M-mode display for the 500 spoke reconstruction. For both Fig. 7c and d, the pericardium (arrows in Fig. 7c & d end-diastole) is best visualized by the corresponding cine videos (supporting video S5) but overall is not as well defined for the 500 spoke reconstructions and an increase in blur and residual artifact is visible as horizontal and vertical lines in the M-modes, respectively. These qualitative results suggest images can be reconstructed using compressed sensing from acquisitions of as few as 750 spokes (acquisition time of ~4 s per slice) without a dramatic decrease in image quality. For reconstructions using fewer than 750 spokes, cardiac dynamics were still visualized, but image degradation including blur, residual aliasing and noise, became more noticeable. Quantitative analysis of undersampled reconstructions is provided below.

**Additional file 5: Video S5.** Reordered cine reconstructions of four retrospectively undersampled data sets (number of reconstructed spokes (N_R_) = 500, 750, 3000). This video corresponds to the same images shown in Fig. 7. Short axis (a & b) and long axis (c & d) views are shown for two different volunteers. Arrows denote the thickness of the myocardium (a), fetal abdominal cavity (b), and pericardium (c & d), features that are degraded with increasing undersampling. (MP4 5145 kb)


**Fig. 8:**
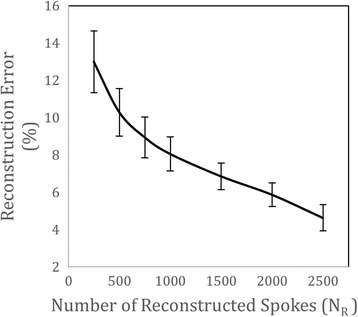
Quantitative evaluation of compressed sensing using retrospectively undersampled human fetal data. Image error as a function of the number of reconstructed spokes (N_R_). Plotted values represent the mean and standard deviation of 96 retrospectively undersampled data sets reconstructed as reordered cine images

Figure 8 plots image error versus number of reconstructed spokes (N_R_) for acquisitions with only in-plane motion (N_xy_ = 96). Plotted values represent the mean over all such acquisitions, with error bars corresponding to the standard deviation. Image error (Fig. 8a), relative to N_R_ = 3000 spokes, decreased monotonically with increasing N_R_ and was approximately 10% for reconstructions using 750 spokes. Recall that reconstructions from 750 spokes were identified as an undersampling limit in Fig. 6. These qualitative and quantitative results are consistent with previous work using compressed sensing to reconstruct images of the fetal heart from Cartesian acquisitions, where reconstructions with an error of approximately 10% were also identified as an undersampling limit [15]. Beyond this level of undersampling, there is a noticeable increase in blur from regularization, residual aliasing, and random noise, which contributes to a rapid increase in image error. There is variability in image error between acquisitions for the same N_R_. However, this is expected based on the range of heart rates exhibited by the different fetal subjects (listed in Table 1) which causes variations amongst the k-space sampling pattern after sorting the acquired spokes by cardiac phase in order to reconstruct reordered cine images [31].

Overall, the qualitative and quantitative evaluations of undersampled acquisitions presented by Figs. 7 and 8 suggest that high resolution dynamic images of the fetal heart can be reconstructed from as few as 750 spokes using compressed sensing, corresponding to an acceleration factor (with respect to the Nyquist sampling criterion) of 16 (acquisition time of ~4 s per slice). These results provide strong validation for the use of compressed sensing to reconstruct images of the fetal heart from undersampled golden angle radial acquisitions and support the proposed strategy for minimizing the effects of through-plane motion through data rejection. In particular, retrospective analysis of slices undergoing visible through-plane motion identified quiescent periods (T_Q_) of 5.5 s in length (mean and standard deviation for all slices containing through-plane motion N_xyz_ = 31). These reconstruction windows correspond to 1140 spokes which is within the acceleration limit outlined above.

## Discussion

In this work, a strategy for reconstructing dynamic images of the fetal heart was developed and evaluated. Golden angle radial datasets were acquired and reconstructed as real-time images. Using the real-time reconstructions, acquisitions with visible through-plane motion were reduced to one or more subsets of data representing quiescent periods. In-plane motion was estimated using registration of the real-time images, and a fetal cardiac gating signal was calculated using metric optimized gating. These motion parameters were used to correct the acquired K-space data and produce high quality reordered cine images of the fetal heart. This approach successfully supressed common sources of motion artifact in fetal cardiac imaging (gross fetal movement, maternal respiration, fetal cardiac motion) and improved temporal resolution and SNR relative to real-time reconstructions of the same data. Furthermore, using compressed sensing, image quality was well preserved up to an acceleration factor of 16 yielding a minimum acceptable acquisition time of 4 s per slice during quiescent through-plane motion.

**Fig. 9:**
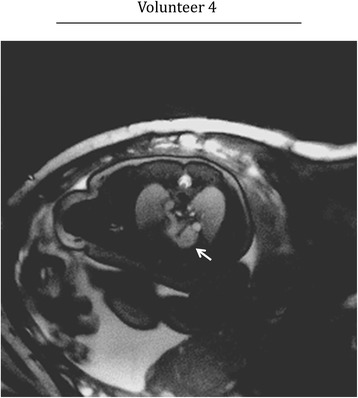
Visualization of congenital heart defect using the current approach. An abnormal three vessel view is shown wherein the ascending aorta (arrow) is larger than the main pulmonary artery. This abnormality indicates tetralogy of Fallot which was confirmed by ultrasound. A cine corresponding to this reconstruction is available as supporting video S6



Potential clinical applications of this work include segmented analysis of fetal cardiac anatomy [7], and quantification of cardiac function [5], both of which are likely to benefit from improved image quality using the current approach. As an example, Fig. 9 depicts a congenital abnormality in a reordered cine image from volunteer 4 with cine videos of this reconstruction provided by supplementary video S6. A three vessel view is shown with the ascending aorta (denoted by an arrow) larger than the main pulmonary artery, in keeping with tetralogy of Fallot. This diagnosis was confirmed by ultrasound. While further evaluation of the impact of image quality on clinical measures of interest was beyond the scope of this work, Fig. 9 helps illustrate the potential of the current approach and further study of specific clinical applications including comparison to ultrasound, the current primary fetal cardiac imaging modality, is warranted.

**Additional file 6: Video S6.** Reordered cine  reconstruction of an abnormal three vessel view. This video corresponds to the image shown in Fig. 9. An arrow denotes the ascending aorta which is larger than the main pulmonary artery. This abnormality indicates tetralogy of Fallot which was confirmed by ultrasound. (MP4 9743 kb)


The primary aims of this study were to evaluate the proposed reconstruction framework for fetal CMR in its ability to supress artifact from motion and radial undersampling. With that in mind, the acquisition time per slice (16 s) and number of slices per cardiac view (10–15) were chosen to provide sufficiently long scans to evaluate motion correction and retrospective undersampling, across a broad range of dynamic fetal cardiac structures. For specific clinical applications, the choice of acquisition time per slice presents an interesting trade-off. Specifically, longer slice acquisitions provide greater opportunity for identifying periods of quiescent motion, ensuring optimal delineation of cardiac anatomy in individual slices. Conversely, shorter slice acquisitions help minimize motion between slices ensuring proper alignment in volumetric reconstructions, but may require repeat measurements if rejection of data corrupted by through-plane motion results in fewer than the minimum number of spokes per image (750) determined above.

The individual components of the proposed reconstruction framework, including radial acquisition, motion correction, metric optimized gating, and compressed sensing, were developed in previous studies of the fetal and post-natal heart. In this current work, we integrated these methods to supress the effects of motion, creating a novel approach to fetal CMR. Additionally, this study presents qualitative and quantitative analysis of motion correction and undersampling in the context of radial fetal CMR data over a range of heart rates and levels of through-plane and in-plane motion. Still, alternative strategies for fetal CMR have been proposed. Our previous work employed a combination of metric optimized gating and compressed sensing to reconstruct reordered cine images from undersampled Cartesian [15] and golden angle radial [5] fetal cardiac data. In these studies, the effects of motion were lessened due to the accelerated acquisition times but no motion correction was performed. van Amerom et al. developed a method for motion-corrected reordered cines by combining frames from real-time k-t SENSE reconstructions of undersampled Cartesian fetal cardiac data [14]. Their study rejected through-plane motion using self gating of maternal respiration, corrected in-plane motion using rigid registration, and resolved fetal cardiac motion by extracting the fetal heart rate from the temporal frequencies of the real-time reconstructions. Chaptinel et al. combined image-based self gating and compressed sensing to reconstruct both real-time and reordered cine images from golden angle radial fetal cardiac data in a manner similar to the proposed framework [6]. Acquisitions were performed under maternal-breath-hold, through-plane motion was visually identified and rejected, but in-plane motion was not corrected. Our current study builds on these promising techniques for fetal CMR to provide a more comprehensive reconstruction framework in the presence of motion. Potential improvements to the current approach are discussed below.

### Motion compensation

In utero datasets were acquired from 7 fetal subjects featuring a range of in-plane displacements (0.3–3.7 mm) and fetal heart rates (119–176 bpm). A total of 150 slices were acquired across all subjects, although 23 slices did not contain cardiac anatomy due to either movement between the slice prescription and acquisition, or due to the acquisition volume being deliberately prescribed to extend beyond the cardiac anatomy to ensure coverage. Using the proposed analysis, it was possible to reconstruct motion-robust cine images of the fetal heart from the remaining 127 slices.

Approximately 21% of the acquired slices contained visible through-plane motion due to maternal respiration or gross fetal movement. Importantly, within each of these slices, at least one period of quiescent motion could be identified. When more than one quiescent period was found for a single slice, each reconstruction provided noticeably different slice positions. This highlights the importance of retrospectively selecting quiescent periods to reject data that would otherwise degrade image quality.

Visual detection of through-plane motion in this study was limited by the prescribed slice thickness of 4 mm. Given the observed range of in-plane displacements, it is likely that mild through-plane motion went undetected in additional slices. Additionally, the identification of quiescent periods was facilitated by the relatively long acquisition time for each slice (15 s). For fetal subjects displaying frequent motion (i.e., at earlier gestational ages), a practical approach may be to acquire slices quickly and repeatedly, without retrospectively reducing datasets. This is similar to approaches used for static imaging of the fetal brain [9–11]. Based on our findings, an acquisition time of approximately 4 s per slice is sufficient to reconstruct cine images without dramatic loss of quality.

In this work, detection of through-plane motion was performed manually. To facilitate clinical applications, automated rejection of data undergoing through-plane motion will be required. While such automation was beyond the scope of this study, previously described methods for outlier rejection in motion correction of fetal images may be applicable [22]. For example, the correlation between real-time frames across the full field-of-view may yield an appropriate signal for identifying regions of through-plane motion [14].

In-plane motion correction was performed using two-dimensional image registration over a region of interest containing the heart and nearby anatomy (i.e., lungs, liver, abdominal wall). Through this process, cardiac motion could affect the calculation of in-plane translational motion. This may be prevented by manually masking the heart; however, inspection of displacement curves (e.g., Figs. 2c, f, i) found little evidence of cardiac effects, with minor jitter attributed to noise and residual aliasing. Conversely, structures undergoing non-rigid in-plane motion may be disrupted by the current registration and correction routine, adversely effecting image quality. For example, a dark fetal limb moving in bright amniotic fluid could produce streaking artifact that obscures the cardiac anatomy and cannot be suppressed with translational motion correction and compressed sensing. Previous studies have successfully employed non-rigid image registration to correct golden angle acquisitions of the post natal heart [19]. Applying non-rigid registration to the fetal heart, which is a small fraction of the total field-of-view, may require careful tuning of the motion correction parameters and manual masking of the fetal heart to prevent deformation of cardiac structures. Such non-rigid motion correction could be incorporated into the proposed reconstruction framework, without changing the overall conclusions of this work.

### Metric optimized gating

Metric optimized gating was used to produce a cardiac gating signal for reordered cine reconstructions. While alternative fetal gating strategies exist in the literature including image based self-gating methods [6, 14] and hardware-based gating using CMR compatible ultrasound [32], metric optimized gating was chosen based on its success in previous studies of both post-natal and fetal hearts [12, 20]. Overall, cardiac gating was not considered a limiting factor in this work. Future development of data-driven or hardware based fetal cardiac gating methods may be integrated easily with the proposed framework.

### Compressed sensing

The effect of undersampling on image quality was assessed by retrospectively undersampling acquisitions that did not contain visible through-plane motion. The reordered cine reconstructions were then evaluated by both visual inspection and image error. Overall, image error decreased with increasing number of spokes and both qualitative and quantitative evaluation of undersampled datasets demonstrated that reordered cine images could be reconstructed from as few at 750 spokes (25 spokes/frame) with minimal image degradation compared to the full datasets acquired in this study of 3000 spokes. With respect to the Nyquist sampling criterion, this represents an achievable acceleration factor of 16 for reordered cine images with a base resolution of 256 and reconstructed to 30 frames [21]. Accelerating beyond a factor of 16 resulted in increased blur and residual aliasing. As a comparison, real-time reconstructions in this work corresponded to an acceleration factor of 27 (15 spokes/frame) and exhibited inferior image quality to the reordered and motion-corrected cine reconstructions, although certain real-time reconstructions were spatially sharper than reordered cine acquisitions without motion correction. Still, major cardiac structures could be identified in both the real-time reconstructions and in reordered cine reconstructions using the smallest tested number of spokes (N_R_ = 250). For cases where acceleration exceeds the proposed limit of 16 (i.e., T_Q_ < 4 s), a greater number of spokes may be used per frame, effectively trading temporal resolution for reconstruction fidelity. Nevertheless, in this study most identified periods of quiescent through-plane motion were within the acceleration limit of 16, providing validation of the proposed framework.

It is worth noting that the largest number of spokes (N_R_ = 3000) used in the undersampling analysis produced reconstructions that were still sub-Nyquist (100 spokes/frame) by a factor of 4 and are therefore not a gold-standard reference. However, given the incoherent nature of aliasing from undersampled golden angle radial data, there are diminishing returns when approaching the Nyquist rate. Thus, the number of spokes acquired per slice in this work was chosen to facilitate multi-slice acquisitions, spanning the fetal heart for both short axis and long axis views, in a reasonable scan time (~5 min), while also providing satisfactory image quality.

Finally, spatial total variation, temporal total variation, and temporal Fourier transform were used for image regularization in the compressed sensing routine used in this work, with higher weighting placed on temporal regularization [15]. As a result, both spatial and temporal blur increased with undersampling factor due to over-regularization as shown in Fig. 8b. Alternative regularization terms such as wavelet, discrete cosine, and low rank may be appropriate for compressed sensing reconstruction of fetal cardiac data and should be explored in order to increase the achievable acceleration factor without image degradation and potentially providing additional improvements to the proposed framework.

### Reconstruction time

Using the proposed framework, approximately 2.5 h were required to reconstruct each slice on a personal computer. To confirm slice prescription and assess fetal activity while the subject was in the scanner, static images (using all acquired spokes) were first reconstructed for each slice. However, reducing reconstruction time of dynamic images is necessary to improve the clinical feasibility of the current approach. The primary contributor to computation time is real time image reconstruction (~2 h) due to the high frame rate required for metric optimized gating. If the fetal heart rate could be measured independent of the real time reconstructions (i.e., K-Space based self-gating, hardware based gating), a lower frame rate could be employed for motion correction to reduce computation time. Additionally, a combination of code optimization and high performance computing should be implemented to reduce reconstruction times and improve the practicality of the current approach in a clinical setting.

## Conclusion

In conclusion, an approach to dynamic CMR of the human fetal heart was developed that is robust to the sources of motion that typically affect fetal CMR. Using golden angle radial acquisitions, motion correction, metric optimized gating, and compressed sensing, high quality reordered cine images were produced and evaluated for varying degrees of motion. This work presents a novel strategy for reconstructing images of the fetal heart and will facilitate future comprehensive examinations of fetal cardiovascular anatomy and physiology in healthy pregnancies and pregnancies affected by cardiovascular diseases including congenital heart disease and placental insufficiency.

## Abbreviations

MOG: Metric Optimized Gating
CMR: Cardiovascular Magnetic Resonance
SNR: Signal-to-noise ratio
